# Higher Stress Hyperglycemia Ratio Is Associated With a Higher Risk of Stroke-Associated Pneumonia

**DOI:** 10.3389/fnut.2022.784114

**Published:** 2022-02-22

**Authors:** Jiejie Tao, Zhishan Hu, Feiling Lou, Junxin Wu, Zijing Wu, Shuang Yang, Xiaofang Jiang, Meihao Wang, Qiqi Huang, Wenwei Ren

**Affiliations:** ^1^Department of Radiology, The First Affiliated Hospital of Wenzhou Medical University, Wenzhou, China; ^2^State Key Laboratory of Cognitive Neuroscience and Learning, Beijing Normal University, Beijing, China; ^3^School of Mental Health, Wenzhou Medical University, Wenzhou, China; ^4^Department of Neurology, The First Affiliated Hospital of Wenzhou Medical University, Wenzhou, China; ^5^Department of Cardiac Care Unit, The First Affiliated Hospital of Wenzhou Medical University, Wenzhou, China

**Keywords:** stroke, pneumonia, glucose, stress hyperglycemia, diabetes

## Abstract

**Objective:**

Stroke-associated pneumonia (SAP) is a frequent complication in stroke patients. This present study aimed to investigate the association between stress hyperglycemia and SAP.

**Methods:**

Patients were screened between February 2013 and August 2020 from the First Affiliated Hospital of Wenzhou Medical University. We divided the blood glucose of the patients at admission by the glycated hemoglobin to calculate the stress hyperglycemia ratio (SHR). Binary logistic regression analysis was used to identify the association between SAP and SHR, with the confounders being controlled. Further, subgroup analyses were separately performed for stroke patients with and without diabetes.

**Results:**

A total of 2,039 patients were finally recruited, of which 533 (26.14%) were diagnosed with SAP. SHR were divided into four quartiles in the logistic regression analysis, the highest SHR quartile (SHR ≥ 1.15) indicated a higher risk of SAP (OR = 1.57; 95% CI = 1.13–2.19, *p* = 0.01) in total patients. In patients without diabetes, the third quantile (SHR = 0.96–1.14) and the highest quantile (SHR ≥ 1.15) were both related to a higher risk of SAP (both *p* < 0.05). However, we did not find such an association in diabetic patients.

**Conclusion:**

SHR was significantly associated with the risk of SAP in patients without diabetes. Adequate attention should be paid to the patients with high SHR levels at admission, especially those without diabetes.

## Introduction

Stroke-associated pneumonia (SAP) is a frequent and serious complication in stroke patients (1). Accumulating studies have observed that SAP was associated with a higher risk of mortality, a longer length of hospitalization, and poorer functional outcomes in stroke survivors independently (2–4). Given the clinical importance of SAP, early identification of SAP is important to improve the prognosis of stroke.

As far as we know, nearly half of patients suffering from stroke may develop stress hyperglycemia (5, 6), which is defined as temporarily increased glycemia in the emergency situation (7). A previous study showed that stress hyperglycemia was associated with a high risk of mortality and recurrence after stroke (6). It should be noted that hyperglycemia was also linked to an increased risk of infection *in vitro* (8) and after surgical procedures (9), which is due to the excessive release of proinflammatory cytokines, such as tumor necrosis factor-α (TNF-α), interleukin-1 (IL-1) and interleukin-6 (IL-6) (10, 11). These elevated cytokines would further interfere with the insulin signaling pathway, reduce insulin production in peripheral tissues, and further increase blood glucose, resulting in a vicious cycle (12). Furthermore, the aforementioned pro-inflammatory molecules were significant contributors to SAP as well (13–16), and the stroke-induced immunosuppression and infection will promote and accelerate the occurrence and development of SAP (17). Thus, stroke patients with a stress hyperglycemia-induced high inflammatory state may be associated with a high risk of SAP, and it was worth exploring the relationship between stress hyperglycemia and SAP.

Many studies adopted the stress hyperglycemia ratio (SHR) to indicate stress hyperglycemia (18–21), which is defined as the ratio of glycated hemoglobin (HbA1c) to blood glucose (21, 22). Moreover, SHR was considered as an independent influencing factor for hemorrhagic transformation in stroke patients (23). In addition, increasing evidence demonstrated that SHR was useful in assessing neurological deterioration and prognosis in patients with ischemic stroke (24), and was related to all-cause death in ischemic stroke patients (25). Moreover, SHR for acute ischemic stroke after intravenous thrombolysis was predictive for worse outcomes (26, 27).

Up to now, the relationship between SHR and SAP was poorly explored. This research aimed to explore the role of SHR in SAP, and we hypothesized that higher SHR may indicate a higher risk of SAP in patients with stroke.

## Materials and Methods

### Study Design and Setting

This study was a retrospective analysis of the patients admitted to the Department of Neurology at the First Affiliated Hospital of Wenzhou Medical University between February 2013 and August 2020. This study was approved by the local Ethics Committee of the First Affiliated Hospital of Wenzhou Medical University. Because this was a retrospective study, written informed consent was waived, and all data were kept anonymous.

### Participants

All the patients were screened from the First Affiliated Hospital of Wenzhou Medical University. The inclusion criterias were: (1) age ≥ 18 years old; (2) onset of stroke within 7 days; (3) acute stroke (including acute ischaemic stroke and intracerebral hemorrhage) verified by a radiological examination [either magnetic resonance imaging (MRI) or cranial computed tomography (CT)]. The exclusion criterias were as follows: (1) preexisting pneumonia before stroke; (2) fever or active infection within 2 weeks before admission; (3) diagnosis of transient ischaemic attack (TIA); (4) data incomplete. The diagnosis of SAP was determined by two well-trained and experienced neurological physicians during the 1st week of hospitalization after the onset of stroke, according to the modified Centers for Disease Control and Prevention criteria for hospital-acquired pneumonia, combining the clinical, laboratory, and radiological examinations (28, 29).

### Data Collection

Data were collected from the electronic medical record system. Demographic variables included age, body mass index (BMI, kg/m^2^), and gender. The medical histories were gathered, including hypertension, coronary heart disease, diabetes mellitus, kidney disease, tuberculosis, chronic hepatitis, and atrial fibrillation. Besides, other clinical characteristics, including cigarette smoking, alcohol consumption, blood pressure, subtypes of stroke, and swallowing function, were recorded as well. The patients' swallowing functions were assessed by the specialists, and thus the diet was determined, including general diet, semi-liquid diet, paste meal, and nasal feeding. The diet was adopted as an indicator of the swallowing function in this study. Furthermore, the National Institutes of Health Stroke Scale (NIHSS) score was also collected, which is a well-validated and reliable scale for evaluating the severity of stroke (30).

Blood samples were carried out after overnight fasting within 24 h of admission to the hospital. We conducted the blood parameters including low-density lipoprotein cholesterol (LDL-C), triglyceride, leukocyte count, serum creatinine (Cr), glucose levels, and HbA1c. Finally, we calculated the stress hyperglycemia ratio by dividing the fasting plasma glucose at admission with HbA1c.

### Study Size

As was shown in Supplementary Figure S1, 2616 patients were screened and 2,039 patients with acute stroke were finally included in this study, of which 533 participants were confirmed as SAP. Meanwhile, 577 patients did not meet the entry criteria and were excluded, of which 220 subjects had a fever or active infection within 2 weeks before admission, 80 had a transient ischemic attack, 67 had pneumonia before the stroke, and 210 had incomplete data.

### Statistical Methods

Continuous variables were displayed as mean ± standard deviation (SD) or median (interquartile range, IQR). A Student's *t*-test (normal distribution) or Mann–Whitney *U*-test (non-normal distribution) was used to compare the continuous variables between the SAP and non-SAP groups. The Chi-square test or Fisher's exact test was used to analyze categorical variables, which were shown as frequencies (percentage). The sample mean approach was conducted to replace missing values. To identify the relationship between SHR and SAP, binary logistic regression analysis was applied with the confounding variables being controlled (including atrial fibrillation, NIHSS score, swallowing function, creatinine, and leukocyte count). SHR was stratified into quartiles ( ≤ 0.84, 0.85–0.95, 0.96–1.14, ≥1.15) in the regression model. Odds ratios (OR) with the corresponding 95% confidence intervals (CIs) were adopted to show the association between the risk factors and SAP. To further explore how diabetes modulated the relationship between clinical presentations and SAP, we conducted binary logistic regression on diabetic and non-diabetic patients. A forest plot was drawn to show the results of the logistic regression analysis.

The significance level was set at *P* < 0.05 (two-tailed). All statistical analyses were performed using SPSS version 21.0 and R (version 4.0.3).

## Results

### Differences in Clinical Presentations Between SAP and Non-SAP Patients

Differences in clinical characteristics between SAP and non-SAP patients were listed in Table 1. Among the total patients, 533 (26.14%) had SAP. These two groups demonstrated no statistical differences in age, gender, BMI, smoking, drinking, hypertension, heart disease, diabetes, kidney disease, tuberculosis, and liver disease (all *P* > 0.05). SHR, leukocyte count, glucose, and creatinine levels were higher in the SAP group than in the non-SAP group (all *P* < 0.01). Significant differences were also found between the groups in the percentage of atrial fibrillation, NIHSS scores, and swallowing function (all *P* < 0.001). Besides, there were no differences between the included and excluded patients in SHR and glucose levels, except for some secondary variables (see Table 2 for details).

**Table 1:** Comparison of characteristics between non-SAP and SAP groups in the total patients.

**Variables**	**Total** **(*n* = 2,039)**	**non-SAP** **(*n* = 1,506)**	**SAP** **(*n* = 533)**	**χ2/*t*/*U***	***P***
Age, Mean ± SD	70.27 ± 11.60	70.18 ± 11.44	70.51 ± 12.08	−0.559	0.576
Gender (male), *n* (%)	1,272 (62.38)	926 (61.49)	346 (64.92)	1.828	0.176
BMI, Mean ± SD	23.85 ± 2.52	23.80 ± 2.53	23.99 ± 2.50	−1.519	0.129
Hypertension, *n* (%)	1,446 (70.92)	1,082 (71.85)	364 (68.29)	2.241	0.134
Heart disease, *n* (%)	280 (13.73)	195 (12.95)	85 (15.95)	2.742	0.098
Diabetes, *n* (%)	612 (30.01)	444 (29.48)	168 (31.52)	0.684	0.408
Kidney disease, *n* (%)	48 (2.35)	33 (2.19)	15 (2.81)	0.421	0.516
Tuberculosis, *n* (%)	21 (1.03)	18 (1.20)	3 (0.56)	0.986	0.321
Liver disease, *n* (%)	38 (1.86)	29 (1.93)	9 (1.69)	0.026	0.872
Atrial fibrillation, *n* (%)	369 (18.10)	225 (14.94)	144 (27.02)	37.926	<0.001^***^
Smoking, *n* (%)	699 (34.28)	511 (33.93)	188 (35.27)	0.258	0.612
Drinking, *n* (%)	617 (30.26)	452 (30.01)	165 (30.96)	0.124	0.724
SBP (mmHg), Mean ± SD	157.17 ± 23.93	157.21 ± 23.94	157.03 ± 23.93	0.152	0.879
DBP (mmHg), Mean ± SD	86.78 ± 22.67	86.75 ± 24.77	86.87 ± 15.27	−0.134	0.893
Stroke type (Hemorrhage), *n* (%)	720 (35.31)	519 (34.46)	201 (37.71)	1.68	0.195
Swallowing function, *n* (%)				379.432	<0.001^***^
General diet	462 (22.66)	419 (27.82)	43 (8.07)		
Semi-liquid diet	873 (42.82)	745 (49.47)	128 (24.02)		
Paste meal	87 (4.27)	59 (3.92)	28 (5.25)		
Nasal feeding	617 (30.26)	283 (18.79)	334 (62.66)		
NIHSS, Median (IQR)	5.00 (2.00, 10.00)	4.00 (2.00, 8.00)	9.00 (5.00, 15.00)	230,768	<0.001^***^
Triglyceride, Mean ± SD	1.56 ± 1.01	1.57 ± 0.93	1.51 ± 1.19	1.153	0.249
LDL_C, Mean ± SD	2.81 ± 0.93	2.82 ± 0.92	2.78 ± 0.95	0.751	0.453
Leukocyte count (10^9^/L), Mean ± SD	7.98 ± 2.70	7.54 ± 2.36	9.23 ± 3.18	−11.176	<0.001^***^
Creatinine (μmol/L), Mean ± SD	77.31 ± 37.89	75.56 ± 34.41	82.23 ± 46.00	−3.058	0.002^**^
Glucose levels (mmol/L), Mean ± SD	6.57 ± 2.74	6.25 ± 2.46	7.48 ± 3.24	−8.006	<0.001^***^
HbA1c (%), Mean ± SD	6.32 ± 1.35	6.31 ± 1.34	6.35 ± 1.40	−0.585	0.558
Glucose/HbA1c ratio, Mean ± SD	1.03 ± 0.32	0.98 ± 0.27	1.17 ± 0.40	−10.27	<0.001^***^

** *P < 0.01;*

*** *P < 0.001. SAP, stroke-associated pneumonia; BMI, body mass index; NIHSS, National Institutes of Health Stroke Scale; LDL-C, low-density lipoprotein cholesterol; SD, standard deviation; IQR, interquartile range; Fisher, Fisher exact test*.

**Table 2:** Comparison of characteristics between included and excluded patients.

**Variables**	**Total (*n* = 2,616)**	**Excluded (*n* = 577)**	**Included (*n* = 2,039)**	**Statistic**	***p***
Age, Mean ± SD	69.59 ± 11.75	67.22 ± 11.95	70.27 ± 11.60	−5.446	<0.001^***^
Gender (male), *n* (%)	1,588 (60.7)	316 (54.77)	1,272 (62.38)	10.623	0.001^**^
BMI, Mean ± SD	25.28 ± 18.8	30.34 ± 39.35	23.85 ± 2.52	3.958	<0.001^***^
Hypertension, *n* (%)	1,880 (71.87)	434 (75.22)	1,446 (70.92)	3.902	0.048^*^
Heart disease, *n* (%)	376 (14.37)	96 (16.64)	280 (13.73)	2.853	0.091
Diabetes, *n* (%)	770 (29.43)	158 (27.38)	612 (30.01)	1.376	0.241
Kidney disease, *n* (%)	64 (2.45)	16 (2.77)	48 (2.35)	0.178	0.673
Tuberculosis, *n* (%)	27 (1.03)	6 (1.04)	21 (1.03)	0	1
Liver disease, *n* (%)	50 (1.91)	12 (2.08)	38 (1.86)	0.026	0.871
Atrial fibrillation, *n* (%)	451 (17.24)	82 (14.21)	369 (18.10)	4.491	0.034^*^
Smoking, *n* (%)	871 (33.3)	172 (29.81)	699 (34.28)	3.851	0.05
Drinking, *n* (%)	775 (29.63)	158 (27.38)	617 (30.26)	1.65	0.199
SBP (mmHg), Mean ± SD	157.23 ± 24.55	157.44 ± 26.63	157.17 ± 23.93	0.225	0.822
DBP (mmHg), Mean ± SD	86.67 ± 21.15	86.28 ± 14.54	86.78 ± 22.67	−0.639	0.523
Stroke type (Hemorrhage), *n* (%)	863 (32.99)	143 (28.77)	720 (35.31)	7.61	0.006^**^
Swallowing function, *n* (%)				23.732	<0.001^***^
General diet	647 (24.73)	185 (32.06)	462 (22.66)		
Semi-liquid diet	1,097 (41.93)	224 (38.82)	873 (42.82)		
Paste meal	115 (4.4)	28 (4.85)	87 (4.27)		
Nasal feeding	757 (28.94)	140 (24.26)	617 (30.26)		
NIHSS, Median (IQR)	5 (2, 9)	2.00 (1.00, 6.00)	5.00 (2.00, 10.00)	402,309.5	<0.001^***^
Triglyceride, Mean ± SD	1.59 ± 1.02	1.70 ± 1.06	1.56 ± 1.01	2.913	0.004^**^
LDL_C, Mean ± SD	2.8 ± 0.92	2.75 ± 0.91	2.81 ± 0.93	−1.331	0.183
Leukocyte count (10^9^/L), Mean ± SD	8.1 ± 2.85	8.49 ± 3.30	7.98 ± 2.70	3.411	<0.001^***^
Creatinine (μmol/L), Mean ± SD	78.31 ± 46.67	81.85 ± 69.22	77.31 ± 37.89	1.515	0.13
Glucose levels (mmol/L), Mean ± SD	6.57 ± 2.69	6.57 ± 2.54	6.57 ± 2.74	−0.026	0.98
HbA1c (%), Mean ± SD	6.34 ± 1.33	6.4 ± 1.24	6.32 ± 1.35	1.386	0.166
Glucose/HbA1c ratio, Mean ± SD	1.03 ± 0.31	1.03 ± 0.30	1.03 ± 0.32	0.402	0.688

* *P < 0.05;*

** *P < 0.01;*

*** *P < 0.001. SAP, stroke-associated pneumonia; BMI, body mass index; NIHSS, National Institutes of Health Stroke Scale; LDL-C, low-density lipoprotein cholesterol; SD, standard deviation; IQR, interquartile range; Fisher, Fisher exact test*.

### Impact of Diabetes on the Relationship Between Clinical Presentations and SAP

Table 3 showed the subgroup analysis of patients stratified by diabetic status. Both the diabetic and non-diabetic patients showed significant differences (SAP vs. non-SAP) in the atrial fibrillation history, NIHSS score, swallowing function, leukocyte count, glucose levels, and SHR (all *P* < 0.001). Additional differences (SAP vs. non-SAP) were found in the non-diabetic patients, including hypertension history, HbA1c, and creatinine (all *P* < 0.05).

**Table 3:** Comparison of characteristics between non-SAP and SAP groups stratified by the diabetic status.

**Variables**	**Non-diabetic group**		***P***	**Diabetic group**		***P***
	**non-SAP (*n* = 1,062)**	**SAP (*n* = 365)**		**non-SAP (*n* = 444)**	**SAP (*n* = 168)**	
Age, Mean ± SD	69.84 ± 11.75	70.25 ± 12.80	0.592	70.99 ± 10.61	71.10 ± 10.33	0.912
Gender (male), *n* (%)	674 (63.47)	240 (65.75)	0.47	252 (56.76)	106 (63.10)	0.184
BMI, Mean ± SD	23.64 ± 2.53	23.81 ± 2.57	0.285	24.19 ± 2.50	24.40 ± 2.30	0.319
Hypertension, *n* (%)	723 (68.08)	227 (62.19)	0.046^*^	359 (80.86)	137 (81.55)	0.937
Heart disease, *n* (%)	129 (12.15)	56 (15.34)	0.14	66 (14.86)	29 (17.26)	0.545
Kidney disease, *n* (%)	16 (1.51)	9 (2.47)	0.33	17 (3.83)	6 (3.57)	1
Tuberculosis, *n* (%)	13 (1.22)	3 (0.82)	0.774	5 (1.13)	0 (0.00)	0.33
Liver disease, *n* (%)	22 (2.07)	7 (1.92)	1	7 (1.58)	2 (1.19)	1
Atrial fibrillation, *n* (%)	166 (15.63)	99 (27.12)	<0.001^***^	59 (13.29)	45 (26.79)	<0.001^***^
Smoking, *n* (%)	374 (35.22)	135 (36.99)	0.585	137 (30.86)	53 (31.55)	0.946
Drinking, *n* (%)	336 (31.64)	120 (32.88)	0.709	116 (26.13)	45 (26.79)	0.95
SBP (mmHg), Mean ± SD	156.41 ± 24.22	156.64 ± 24.29	0.872	159.14 ± 23.18	157.87 ± 23.18	0.545
DBP (mmHg), Mean ± SD	87.39 ± 28.16	87.33 ± 15.68	0.961	85.20 ± 13.45	85.86 ± 14.34	0.606
Stroke type (Hemorrhage), *n* (%)	410 (38.61)	157 (43.01)	0.155	109 (24.55)	44 (26.19)	0.754
Swallowing function, *n* (%)			<0.001^***^			<0.001^***^
General diet	292 (27.50)	22 (6.03)		127 (28.60)	21 (12.50)	
Semi-liquid diet	539 (50.75)	92 (25.21)		206 (46.40)	36 (21.43)	
Paste meal	42 (3.95)	23 (6.30)		17 (3.83)	5 (2.98)	
Nasal feeding	189 (17.80)	228 (62.47)		94 (21.17)	106 (63.10)	
NIHSS, Median (IQR)	4.00 (2.00, 8.00)	10.00 (5.00, 16.00)	<0.001^***^	4.00 (2.00, 8.00)	9.00 (4.00, 13.00)	<0.001^***^
Triglyceride, Mean ± SD	1.47 ± 0.85	1.36 ± 0.97	0.06	1.83 ± 1.05	1.83 ± 1.53	0.987
LDL_C, Mean ± SD	2.86 ± 0.90	2.82 ± 0.93	0.484	2.73 ± 0.95	2.71 ± 1.00	0.822
Leukocyte count (10^9^/L), Mean ± SD	7.40 ± 2.24	9.27 ± 3.27	<0.001^***^	7.88 ± 2.60	9.12 ± 2.97	<0.001^***^
Creatinine (μmol/L), Mean ± SD	75.05 ± 32.43	83.06 ± 49.06	0.004^**^	76.80 ± 38.76	80.44 ± 38.61	0.3
Glucose levels (mmol/L), Mean ± SD	5.43 ± 1.34	6.33 ± 1.90	<0.001^***^	8.22 ± 3.28	9.99 ± 4.03	<0.001^***^
HbA1c (%), Mean ± SD	5.69 ± 0.38	5.64 ± 0.39	0.019^*^	7.78 ± 1.62	7.89 ± 1.56	0.427
Glucose/HbA1c ratio, Mean ± SD	0.95 ± 0.23	1.12 ± 0.33	<0.001^***^	1.05 ± 0.33	1.28 ± 0.50	<0.001^***^

* *P <0.05;*

** *P <0.01;*

*** *P <0.001. SAP, stroke-associated pneumonia; BMI, body mass index; NIHSS, National Institutes of Health Stroke Scale; LDL-C, low-density lipoprotein cholesterol; SD, standard deviation; IQR, interquartile range; Fisher, Fisher exact test*.

### Associations Between SHR and SAP

**Figure 1:**
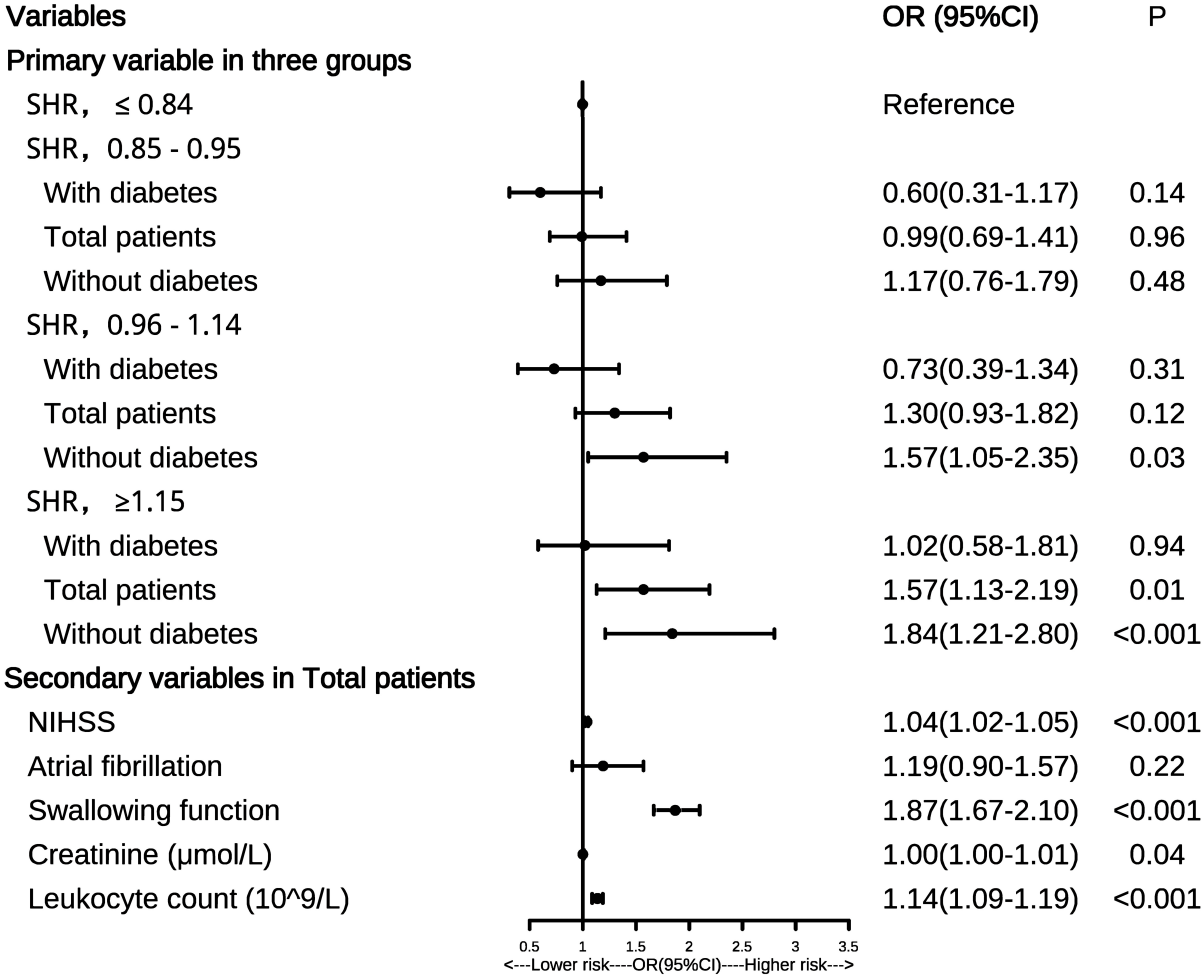
Forest plot of odds ratios for SAP. NIHSS, National Institutes of Health Stroke Scale; SAP, stroke-associated pneumonia; OR, odds ratio; 95%CI, 95% confidence interval.

The forest plot in Figure 1 showed that higher SHR levels (SHR ≥ 1.15) were associated with a higher risk of SAP both in the total patients (*P* = 0.01) and those without diabetes (*P* < 0.001), with the first quantile (SHR ≤ 0.84) being the reference. For patients without diabetes, the third quantile (SHR = 0.96–1.14) and the highest quantile (SHR ≥ 1.15) were both associated with a higher risk of SAP than the first quantile (both *P* < 0.05). However, similar results were not found in patients with diabetes (all *P* > 0.05).

We also found that the secondary variables including NIHSS score, swallowing function, leukocyte count, and creatinine were all risk factors for SAP in the total patients (all *P* < 0.05), which was shown in the forest plot. Similar results were also observed in patients without diabetes (all *P* < 0.05, data not shown). In patients with diabetes, only NIHSS score and swallowing function were risk factors for SAP (both *P* < 0.05, data not shown).

## Discussion

To our knowledge, the current study is the first study describing the relationship between SHR and SAP. There were two main findings of this study. Firstly, we found that higher SHR levels were significantly associated with an increased risk of SAP in patients with stroke. Secondly, this effect mainly existed in patients without diabetes.

Prior studies have reported the worse outcomes of stroke patients with comorbid stress hyperglycemia (6, 23–25). However, there was a lack of consensus on whether stress hyperglycemia deteriorated the severity of SAP. Previous studies showed that hyperglycemia was associated with community-acquired pneumonia (31, 32). In this study, we found that high SHR levels (SHR ≥ 1.15) were also significantly associated with SAP. The mechanism underlying the SHR and SAP was still unknown. As is known to all, hyperglycemia in the context of acute diseases is an adaptive response, which provides energy for the central nervous system, immune system, and various organs and increases the survival opportunity of the host to a certain extent (33). However, stress hyperglycemia also has some harmful effects. Specifically, glucose was identified as a pro-inflammatory mediator in animal and human studies (34). Meanwhile, hyperglycemia can promote the production of inflammatory cytokines, inflammatory processes, and insulin resistance (34). In addition, it can decrease vascular endothelial nitric oxide and promote vasoconstriction, and in turn lead to abnormal organ perfusion (34, 35). Moreover, it also disrupts the immune system, directly inhibiting the function of immunoglobulin, T lymphocytes, and complement, and further increasing the risk of infection (34, 35). Hyperglycemia was also shown to be associated with lower bacterial clearance and higher infection-related mortality in diabetic animal models (36–38). In addition, stroke-induced immunosuppression can be observed in stroke patients, which is a systemic anti-inflammatory response (39). Studies had also found that SAP was closely related to the immunosuppression syndrome caused by stroke (40). Immunosuppression was associated with an excess of glucose, and as mentioned earlier, a hyperglycemic state increased the production of reactive oxygen species by immune cells, leading to oxidative stress and the promotion of proinflammatory cytokine cascades (34). This excessive inflammatory response might deplete the immune system, and at the same time, hyperglycemia in turn could suppress immunoglobulins, T lymphocytes, and complement (34, 40). These factors may ultimately lead to the suppression of systemic immune function, thereby predisposing patients to stroke-induced immunodepression syndrome and stroke-associated infections (40).

Furthermore, we found that the correlation between SHR and SAP was only found in the total and non-diabetic patients, but not diabetic patients. Similar results were also found in previous studies related to brain injury and stroke (24, 41). The mechanism of this issue remains obscure. One possible reason was that acute hyperglycemia could promote oxidative stress, whereas chronic hyperglycemia could induce antioxidant defenses in tissues and cells (42). Therefore, diabetes may increase antioxidant defenses, which can protect tissues from oxidative stress caused by acute hyperglycemia, and thus attenuating inflammation resulting from oxidative stress (43, 44). Future research is needed to explore the underlying mechanism of this phenomenon, which might help to further reveal the pathophysiological mechanism of diabetes development.

Moreover, we found that the secondary variables, including NIHSS score, swallowing function, and leukocyte count, were all risk factors for SAP. Growing evidence had shown that higher NIHSS scores, dysphagia, as well as elevated leukocyte count, could contribute to an increased risk of SAP (45–49). Our study further demonstrated these points.

This study has several limitations. First, this study was limited by the retrospective nature of the analysis, which relied heavily on discharge medical diagnosis coding. Second, this study was a cross-sectional study and could not be done to explore causality, and patients were recruited over a long time range from 2013 to 2020, which may lead to a timely bias because the characteristics of these patients may differ in different years. Third, differences were found in some variables such as age and gender between the included and excluded patients, which may lead to selection bias to some extent. Forth, blood glucose levels are prone to fluctuation, which may have an impact on the results. Fifth, only Chinese stroke patients were included in the current study. Therefore, the findings here may not be generalized to other populations.

## Conclusion

In conclusion, SHR was significantly associated with the risk of SAP in patients without diabetes. Adequate attention should be paid to the patients with high SHR levels at admission, especially those without diabetes.

## Data Availability Statement

The original contributions presented in the study are included in the article/Supplementary Material, further inquiries can be directed to the corresponding author/s.

## Ethics Statement

The studies involving human participants were reviewed and approved by Ethics Committee of The First Affiliated Hospital of Wenzhou Medical University. Written informed consent was not required for the current study in accordance with the local legislation and institutional requirements.

## Author Contributions

WR and QH: conceived and designed the study. WR, ZW, JW, XJ, and JT: made substantial contributions to data analysis and interpretation. JT, ZH, and FL: drafted the manuscript. WR, SY, QH, JT, ZH, and MW: reviewed and gave final approval of the version to be published. All authors have read and approved the final manuscript.

## Funding

This work was supported by the National Natural Science Foundation of China (62007002) and the China Postdoctoral Science Foundation (2020M670189).

## Conflict of Interest

The authors declare that the research was conducted in the absence of any commercial or financial relationships that could be construed as a potential conflict of interest.

## Publisher's Note

All claims expressed in this article are solely those of the authors and do not necessarily represent those of their affiliated organizations, or those of the publisher, the editors and the reviewers. Any product that may be evaluated in this article, or claim that may be made by its manufacturer, is not guaranteed or endorsed by the publisher.
