# Supplementary figures and images for: Higher Stress Hyperglycemia Ratio Is Associated With a Higher Risk of Stroke-Associated Pneumonia
Source: Front Nutr. 2022 Feb 22;9:784114. doi: 10.3389/fnut.2022.784114 (PMC8902157; doi:10.3389/fnut.2022.784114)

**Figure S1.** The flow of participants. **Abbreviation:** SAP: stroke-associated pneumonia.

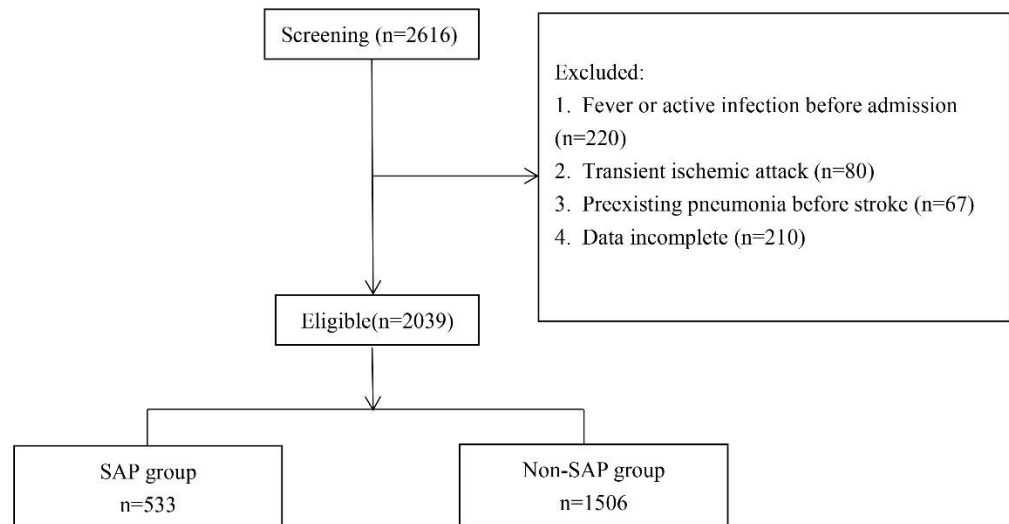

Supplement: Supplementary file 1 [file Image_1.pdf]
